# Inferring Unseen Causes: Developmental and Evolutionary Origins

**DOI:** 10.3389/fpsyg.2020.00872

**Published:** 2020-05-06

**Authors:** Zeynep Civelek, Josep Call, Amanda M. Seed

**Affiliations:** School of Psychology and Neuroscience, University of St Andrews, St Andrews, United Kingdom

**Keywords:** causal reasoning, hidden causes, temporal order, pre-schoolers, chimpanzees

## Abstract

Human adults can infer unseen causes because they represent the events around them in terms of their underlying causal mechanisms. It has been argued that young preschoolers can also make causal inferences from an early age, but whether or not non-human apes can go beyond associative learning when exploiting causality is controversial. However, much of the developmental research to date has focused on fully-perceivable causal relations or highlighted the existence of a causal relationship verbally and these were found to scaffold young children’s abilities. We examined inferences about unseen causes in children and chimpanzees in the absence of linguistic cues. Children (*N* = 129, aged 3–6 years) and zoo-living chimpanzees (*N* = 11, aged 7–41 years) were presented with an event in which a reward was dropped through an opaque forked-tube into one of two cups. An auditory cue signaled which of the cups contained the reward. In the causal condition, the cue followed the dropping event, making it plausible that the sound was caused by the reward falling into the cup; and in the arbitrary condition, the cue preceded the dropping event, making the relation arbitrary. By 4-years of age, children performed better in the causal condition than the arbitrary one, suggesting that they engaged in reasoning. A follow-up experiment ruled out a simpler associative learning explanation. Chimpanzees and 3-year-olds performed at chance in both conditions. These groups’ performance did not improve in a simplified version of the task involving shaken boxes; however, the use of causal language helped 3-year-olds. The failure of chimpanzees could reflect limitations in reasoning about unseen causes or a more general difficulty with auditory discrimination learning.

## Introduction

In life and also in science, much of the evidence we get for causal relations is indirect. We can infer the existence and nature of a cause for an event despite not witnessing it directly: if it is hidden from our perspective, or if it is not perceivable by the senses. Our inferences can range from identifying the cause of a crashing sound coming from the kitchen (the wooden cutting board or the metal pot falling on the floor) to the causes of global warming (anthropogenic impact on the greenhouse effect). But how do we do this? Bullock et al. (1982) suggest that we use the principles of determinism, priority and mechanism: We assume that there is a causal structure to the world (i.e., that events typically have causes); that these structures are unidirectional (i.e., causes come before their effects) and that events are underpinned by a causal mechanism of some kind. Using these principles, and our prior knowledge with regards to specific relations, we can work our way from effects to detect likely causes. This is an extraordinary ability that frees us from relying on what can be directly perceived, allows us to make predictions about the future, and intervene to bring about desirable outcomes.

However, we can also learn regular covariations in spatiotemporal contiguity, which allow us to exploit a causal pattern even if we do not theorize about the generative mechanism (Shanks and Dickinson, 1987). If two events occur repeatedly under close spatiotemporal proximity, we form associative links between them. Later when one of the cues occur, the other can be predicted without any reference to the causal mechanism involved, indeed, without any explicit awareness of the relationship at all (Reber, 1989). Conversely, we can learn a great deal about unseen causal relations without any direct experience: from others’ explicit testimony or implicit linguistic cues to causality (Harris and Koenig, 2006; Gelman, 2009). We may even learn about causal relations we may not have learnt otherwise (e.g., “The gravitational attraction of the moon causes tides”). These three alternative routes to exploiting causal relations in the world (association, theory-building and testimony) are not mutually exclusive, as adults we make us of all of them, and they interact in important ways.

What are the origins of these abilities in human development and over human evolution? There is good evidence that statistical or associative learning is present early in infancy (Aslin et al., 1998; Kirkham et al., 2002), and that this ability is shared with a great many other species. It is similarly uncontroversial that learning from testimony is a route available to children once they learn language, and unique to our species. However, when it comes to going beyond the data to reason about causal mechanisms there is more controversy both in developmental and comparative psychology (Penn and Povinelli, 2007; Bonawitz et al., 2010; Seed et al., 2011). Some researchers have suggested that humans have a natural tendency to explain the events they observe in terms of causal theories from very early in life (Bullock et al., 1982; Gopnik and Wellman, 2012). If this is the case, it is plausible that we share this ability with our closest primate relatives, and possibly other species (Seed et al., 2011; Völter and Call, 2017). Alternatively, others contend that causal thinking in early childhood might not be well-characterized by the notion of “theories all the way down” (Carey and Spelke, 1996). Instead children’s thinking about causation may only approximate scientific thinking later in development, due in part to input from others with the development of language. If this is the case, we may not expect to find causal reasoning in non-human primates. Penn and Povinelli (2007) have argued that there is no evidence non-human animals represent causality as such.

While tackling these questions empirically, one issue common to the comparative and developmental literature concerns distinguishing causal reasoning (based on representations of causal mechanism) from associative learning (making predictions in the absence of these representations), since events that are causally linked tend to co-occur. From a developmental perspective alone, a second issue concerns teasing apart the role of causal language and reasoning since children can use both to solve causal problems. We have two aims in this paper: (1) to further explore children’s inferences about unseen causes in the absence of linguistic cues to causality, and (2) to use the same paradigm to explore this ability in our closest relatives, chimpanzees.

There is substantial research suggesting that preschool children take unseen causal relations into account when explaining natural phenomena such as light (Bullock et al., 1982), wind (Shultz, 1982), electricity (Buchanan and Sobel, 2011), and contamination by germs (Legare et al., 2009). However, it is difficult to isolate the route to causal knowledge in cases that involve familiar events such as these. Children may have extensive prior experience with lights and blowing candles which may lead to forming associative links or may have been explicitly taught by adults about how “germs cause disease.” Indeed, younger preschoolers who supposedly did not have extensive experience with wires and electricity, failed to reason about these relations and made decisions based on covariation information instead (Buchanan and Sobel, 2011). They were only able to solve the problem when it involved more familiar batteries. Although it is possible that experience leads to extracting abstract causal information, it may also lead to learning arbitrary associations (e.g., when there are batteries inside, the toy works).

A way to address this issue has been to present preschoolers with novel and arbitrary causal structures. As adults and scientists, when the evidence we get does not fit with our prior knowledge or expectations, we infer unseen causes or confounding variables. In order to test if children reasoned in the same way, children were first trained on a novel causal structure (e.g., puppets moving in a certain way), and then saw evidence that was inconsistent with their training (Gopnik et al., 2004; Schulz and Sommerville, 2006; Schulz et al., 2008). When children were asked to make predictions about the cause of this inconsistent event, they were more likely to say that an unseen cause (i.e., “something else”) was responsible. Children also displayed an ability to imagine the effect of a hidden cause in a series of experiments by Siegel et al. (2014). They were able to select boxes to shake that would yield unambiguous data (e.g., if their task was to locate a hard object, they chose to pair it with a soft object rather than another hard object). However, in these studies the existence of a cause and the possibility that it might be unseen was provided in the framing of the task by the experimenter so the children did not have to infer it from the evidence alone. For instance, the experimenter asked “Why are the puppets moving together? Is it X, Y or something else?”

Overall, the evidence suggests that by 4 years of age children can successfully detect the presence of an unseen cause and make inferences about their nature; but the potential impact of others’ verbal testimony on their abilities has not been explored to date. Gelman (2009) argued that children are not “lone scientists”: they get much needed input from adults around them. Linguistic framing can help children to specify a causal relation by testifying that the covariations they see are indeed causal; and the use of same wording can point to the commonalities between an observed action and agent’s action (as in intervention studies: “The block makes it go. Can you make it go?”). Indeed, there is accumulating evidence that the use of causal framing can impact children’s propensity to make causal inferences from directly perceived and indirect evidence (Sobel and Sommerville, 2009; Bonawitz et al., 2010; Butler and Markman, 2012; Lane and Shafto, 2017).

One possibility is that verbal framing merely highlights the problem for children: making the task more sensitive to their theory construction ability by reducing peripheral demands such as the need to focus attention (Sobel and Sommerville, 2009). Another possibility is that without the verbal framing younger children are yet to develop some of the fundamental cognitive components needed to construct a causal explanation from evidence alone. The difficulty with using never-seen-before causal relationships is that some training or explanation is necessary for children to have the required background information to make inferences. While the nature of the instructions have been varied, they are rarely excluded. The verbal framing may simplify the task for older children, equally, it may make the test unsuitable for younger children such as 2–3 year-olds if they lack sufficient verbal ability to follow the instructions. We therefore designed a paradigm with minimal language requirements to explore this issue. We also intended to use this paradigm to make comparisons between children and non-human primates. This line of evidence could be very informative in establishing the degree to which human scientific thinking is grounded in skills we share with our closest relatives, or is rather a skill that requires cultural input over development to emerge, and verbal input to elicit in younger children.

Whether or not our closest relatives, chimpanzees, engage in causal reasoning is a controversial issue in comparative psychology. Some authors propose that causal reasoning is a uniquely human ability; and chimpanzees either learn associatively or they rely on generalizations based on the surface appearance of objects alone to solve problems (Penn and Povinelli, 2007; Penn et al., 2008; Bonawitz et al., 2010). Limitations in performance in some tasks designed to probe the causal reasoning abilities of great apes would seem to support this interpretation (Köhler, 1925; Limongelli et al., 1995; Povinelli, 2000; Call, 2007). In contrast to Penn and Povinelli (2007), Seed et al. (2011) proposed that non-human great apes can make use of causal information from events happening around them if the testing situation does not overload other cognitive resources. It could be shown that they did not rely solely on the available sensory information to learn associations. However, it has been a challenge to decisively distinguish associative learning from causal reasoning.

One of the most promising ways to resolve this issue has been to compare how non-human primates (and other animals, such as corvids and dogs) make inferences about the location of food in two contexts, either: (a) the evidence is caused by the food or (b) the evidence co-varies with the presence of food but the relation is arbitrary (reviewed in Seed and Mayer, 2017; Völter and Call, 2017). Great apes successfully used indirect evidence to locate food in a number of studies: in the form of auditory cues coming from shaken cups (Call, 2004), the visible effect of weight (Hanus and Call, 2008); and visible traces or trails (Völter and Call, 2014). In the critical comparison conditions, in which the relationship between a similar cue and the food location was arbitrary rather than causal, apes did not find the food (for example, if the experimenter played the recording of the rattling sound over the baited cup, Call, 2004). Taken together these studies imply that apes are capable of causal reasoning about unseen causes.

However, the comparability of the arbitrary conditions to the causal ones were criticized. For example, Penn and Povinelli (2007) point out that the “recorded sound” control of the shaken cups study was not identical to the sound the shaken cup made. They further argued that the results could still be explained by associative learning if subjects had used the combination of shaking motion and rattling sound as a discriminative cue for locating food. Overall, the comparability of the experimental and control conditions in terms of different feedback (e.g., auditory) poses a challenge for distinguishing causal reasoning from associative learning.

The task presented in this study was designed to address some of the empirical challenges raised above by reducing verbal requirements and implementing robust controls for associative learning. In the “causal condition,” a ball containing a reward was dropped into a forked tube, and could be found in one of two cups at the bottom. After the ball was dropped, participants heard either a *ding* or a *clack* sound. After a few trials, subjects were expected to learn that when they heard a *ding*, the ball would be in one cup and when it was a *clack*, the ball would be in the other one. If subjects succeed in this condition, it might mean that they reasoned about the underlying causal structure (the ball hitting the different boxes caused different sounds) or that they simply associated the sound with the side (if *ding*, choose right). In order to distinguish between these two possibilities, in the “arbitrary condition” the order of events was reversed: participants first heard a *ding* or a *clack* sound, and then the ball was dropped into the forked tube. Although the sounds were still predictive of the location of the ball (if *ding*, choose right), the relationship was now arbitrary. Critically, the two conditions were equivalent from an associative learning perspective since the stimuli involved in both conditions were exactly the same and the only difference was the order of events. However, if participants reason about unseen causes, they are expected to do better in the “causal condition” where there is a plausible causal structure than in the “arbitrary condition.”

In previous studies, we have found such differences between causal and arbitrary conditions in children between the ages of 3 and 5, when dealing with directly perceivable events such as choosing an appropriate tool or an unobstructed path for extracting a reward (Mayer et al., 2014; Seed and Call, 2014). However, such performance differences are not apparent in older children, probably because 6-year olds are capable of interpreting arbitrary cues as symbolic communication to solve a problem (DeLoache, 2004; Seed et al., 2011; Mayer et al., 2014). We therefore focused on the 3–6-year-olds in this study. By 3-years of age children expect causes to precede their effects (Bullock and Gelman, 1979; Rankin and McCormack, 2013) so we predicted that by this age children should perform at above chance levels in the causal condition if they reasoned causally, and by 6-years they should be above chance in both conditions.

## Experiment 1: Children

### Methods

#### Participants

Three-to-six-year-old children (*N* = 129) were tested in different locations in Scotland. There were 65 children in the causal condition and 64 in the arbitrary condition. Age and sex were split roughly equally in the two conditions (Table 1). Twenty-three additional children that were tested were excluded from the study due to experimenter or apparatus error (7), parental interference (3), discovery of the trick about the box (4) and refusal to complete the task (9). All the children studies reported in this paper were ethically approved by University of St Andrews Teaching and Research Ethics Committee and informed consent were taken from parents/guardians.

**TABLE 1:** Age, sex, and mean/median performances of children in Experiments 1, 2, 4, and 5.

	*N* (females)	Mean age	Mean/Median performance	*SD*
**Experiment 1**				
3-year-olds				
Causal	16 (8)	3.6	0.45	0.50
Arbitrary	16 (8)	3.4	0.50	0.50
4-year-olds				
Causal	16 (8)	4.5	**0.62**	0.48
Arbitrary	16 (7)	4.4	0.50	0.50
5-year-olds				
Causal	16 (8)	5.4	0.55	0.49
Arbitrary	16 (8)	5.4	0.48	0.50
6-year-olds				
Causal	17 (8)	6.4	**0.60**	0.49
Arbitrary	16 (8)	6.3	**0.60**	0.49
**Experiment 2**				
4–5-year-olds				
Causal	20 (9)	4.7	**0.61**	0.49
Arbitrary	20 (10)	4.6	**0.62**	0.49
**Experiment 4**				
3-year-olds	16 (8)	3.5	0.54	0.50
4-year-olds	16 (9)	4.5	**0.69**	0.46
5-year-olds	16 (8)	5.4	**0.83**	0.38
**Experiment 5**				
3-year-olds	28 (14)	3.7	**0.67**	0.47

*Numbers in bold represent means that are significantly different from chance (p < 0.05) according to a Wilcoxon signed rank test (Experiment 1) and one sample t-tests (Experiments 2, 4, and 5).*

#### Materials

##### Transparent training box

**FIGURE 1:**
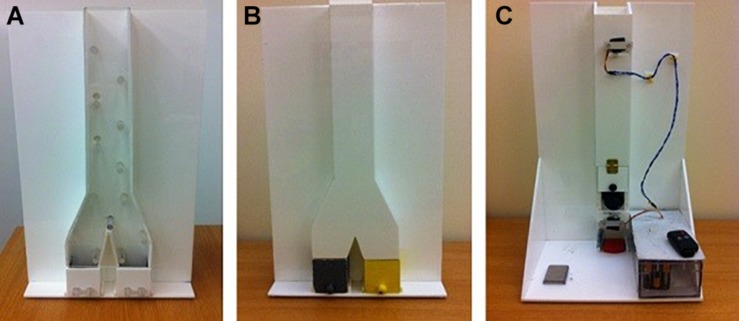
Transparent training box **(A)**, opaque testing box **(B),** and the back of the opaque testing box **(C)** used in Experiments 1 and 3.

The training apparatus was a forked chute made from clear acrylic (Figure 1). The middle singular channel (30 × 6.5 × 5cm) was forked into two channels. Directly at the bottom of the channels there were two white acrylic boxes (2.5 cm apart). The channels were mounted on a white acrylic back panel (30 × 49 cm); and a base panel (25 × 30 cm) to stand. They contained pegs that were 7.5 cm apart from each other on both sides. The pegs were designed to slow down the fall of the ball and to make sounds so that subjects could easily follow the ball’s trajectory. A peg positioned right above the fork could be moved to the either side from behind the back panel. It enabled the experimenter to control which side the ball would fall in a trial.

##### Opaque testing box

The testing apparatus had the same measurements as the training box but the channels were opaque. The boxes at the bottom of the channels were spray-painted, one yellow and one gray, using Plastikote stone-textured paint (Figure 1). In the testing apparatus, the back panel concealed two additional elements which, unbeknownst to the participant, controlled the falling of the ball through the apparatus and the production of the sound cues.

First, there was a middle singular channel (30 × 10 cm) into which the dropped ball would fall, hitting pegs along the way, and land noiselessly on a piece of foam. Below this channel was a shorter one (6.5 cm) in which a second ball was held and could be released onto a noise-making block (wooden or metal). This block could be exchanged by the experimenter depending on the trial. These two components were combined through the action of two small motors which controlled the rotation of small plastic supports that held the two balls in place. When the motors were switched on by a remote, the plastic supports would rotate, releasing the two balls according to a precise timing. The two buttons on the remote controlled the order in which the motors would activate. In the *causal condition*, the motor at the top would operate first and let the ball dropped into the apparatus by the experimenter, go down the channel hitting the pegs, and then the motor at the bottom would release the second ball to fall onto the metal/wooden piece positioned by the experimenter. The intended illusion was that the ball had fallen down the channel into one of the two boxes and made a distinct sound. In the *arbitrary condition*, the activation of the motors was reversed. The second support moved first to release the ball on the metal/wooden piece, and then the experimenter dropped the ball in time for the first support to rotate and let the ball fall down the channel with the pegs. The time interval between the activation of the two motors copied the actual time it would take the ball to fall in reality and was the same in both conditions. In the causal condition it appeared as a single event sequence. The electronic card that controlled the motors was concealed in a box behind the apparatus (Figure 1). The reason for creating the illusion rather than using a real event sequence was that: (1) no local sound cues were given to locate the ball; and (2) the order of the cues could be reversed in the arbitrary condition while keeping everything else about the stimuli exactly the same.

The balls were made of thermoplastic (1.60 cm in diameter) and contained a hole in the middle where the reward could be put.

#### Procedure

##### Training phase

The experiment started with the transparent training box. The experimenter introduced the task saying; “In this game, I will put a sticker in the ball and then I will drop the ball from here (the top opening). It will roll down to one of these boxes (points to the boxes at the bottom). If you find the ball, you will win the sticker. Ready?” The experimenter then dropped the ball and the child could watch the entire trajectory of the ball until it came to rest, hidden, in one of the boxes. The child then pointed to or opened the box that she/he thought the ball was in. Once the child made a choice, the other box was also opened to show the content. Transparent training ended after five consecutive successes or ten trials in total.

##### Test phase

After the training, the experimenter said “This game was too easy for you! Shall we make it more fun?” and brought out the opaque testing box. Then introduced the task to the children; “The game is the same. I will put a sticker in the ball and drop the ball from here. If you can find the ball, you will win the sticker. You cannot see inside the box anymore, but there is still a way to find the ball in the correct box! Do you want to try?” Before each trial, the experimenter prepared the apparatus behind a barrier by putting a ball with a sticker inside into one of the boxes at the bottom, placing another ball on the support attached to the motor just above the metal/wood piece and holding another in her hand for the child to see. The metal and wood pieces were interchanged in between trials and the remote that controlled the events rested behind the apparatus.

In the *causal condition*, the experimenter pressed the causal-order button on the remote while dropping the ball. From the participant’s perspective, they would see the experimenter drop a ball into the apparatus, follow the trajectory of the fall due to the pegs inside the middle channel and then hear a metallic or wooden sound.

In the *arbitrary condition*, the experimenter pressed the arbitrary-order button on the remote. The participant would first hear a metallic/wooden sound and then see the experimenter drop the ball into the apparatus and follow the trajectory of the fall due to the pegs.

If the child found the ball, the experimenter said “Well done! You won a sticker!” removed the other box to show that it was empty and prepared for the next trial. If the child did not find the ball, the experimenter said “Oh no! It was here (opening the other box). Let’s do it again!” In total children got 20 testing trials which lasted about 15 min. For a given participant the position of the yellow and gray boxes at the end of the channels stayed the same over the 20 trials (e.g., the yellow box on the left was associated with a *ding*, and the gray box in the right was associated with a *clack*), but between subjects the pairing of the color of the box and the sound were randomized. The ball was placed in each box 10 times in a random fashion but never in the same box more than twice in succession.

##### Open ended question

At the end of the task, the experimenter asked children; “How did you decide which box to choose?” If children did not reply, the experimenter elaborated “Sometimes the ball was in the gray one and sometimes in the yellow one. How did you know where the ball was?” Other than 15 missing explanations (first 10 participants were not asked because it was not initially planned in the study design and 5 other participants had to leave immediately after testing), all children responded to the question.

#### Scoring and Analysis

The first choice of the subjects was scored as their response in all of the experiments. All trials were scored live by the experimenter as correct or incorrect and were also videotaped. A second examiner coded 20% of the videos for reliability, *Kappa* = 0.97 (95% CI [0.95, 0.99], *p* < 0.001. The mistakes that were found by the second coder were corrected and all the videos were recoded from the video once again to check for other potential mistakes (none were found). The data for this study can be found at Supplementary Table S2.

We specified generalized linear mixed models (GLMM; Baayen, 2008) with binomial error structure and logit link function using the function glmer of the R-package lme4 (Bates et al., 2015) for all of our analyses in this paper. In Experiment 1, our full model comprised of condition (causal/arbitrary), age, and their interaction; trial number, and sex as fixed effects. Subject ID and the side of the boxes were included as random effects. In order to keep type-1 error rates at the nominal level of 5%, we included random slopes of trial number within subject ID, but left out the correlation parameters between random intercepts and random slopes terms (Schielzeth and Forstmeier, 2009; Barr et al., 2013). We compared the full model to a null model which included only the random effects using a likelihood ratio test.

The model stability was assessed by excluding individual cases one at a time and comparing the estimates with those derived from a model with the full data set. The model was stable with regards to the fixed effects. We checked whether the variability was greater than expected (overdispersion) and found that it was not an issue with regards to the final model (dispersion parameter: 0.95). Finally, variance inflation factors (VIF) were calculated using the function vif of the R-package car and it did not indicate collinearity to be an issue.

The data was not normally distributed so non-parametric Wilcoxon signed-rank tests were used to examine whether children’s performance was significantly different from chance level (*p* = 0.05) in different conditions and age groups. Children who chose one side 16 or more times were counted as side biased according to a two-tailed binomial test (*p* = 0.004). Chi-square tests were used to explore the relationship between side bias, condition and age.

Children’s responses to the open-ended questions were categorized into five types of explanations (*N* = 114) using the relevant categories from Legare et al. (2010). The first category, “No explanation,” consisted of children who could/did not provide a verbal strategy (e.g., pointed to the boxes, said “yellow/gray one”). The second category was “Don’t know,” which consisted of children who said they did not know how to find the ball and they were just guessing. The third category was “Non-causal strategies” that referred to a solution based on a non-causal feature or pattern (e.g., the ball alternated right-left-right-left, “because of the colors”). The fourth category was “Causal explanations that were wrong” (e.g., “I followed the noises into the boxes,” “The box wiggled a bit when the ball fell into it”). And the last category was “Referring to different sounds/materials” which showed an understanding of the true causal structure (e.g., “They made two different sounds”). A second examiner categorized children’s answers into these five different types of explanations. There was a high agreement between the two coders, *Kappa* = 0.86 [95% CI, 0.80, 0.93], *p* < 0.001 and it rose to *Kappa* = 0.96 [95% CI, 0.92, 0.99], *p* < 0.001 after further discussions. The disagreements were due to some responses that could be categorized either as category one or two (e.g., “Don’t know” and points to the boxes). We decided to include them in “no explanation” category as they were mostly pointing gestures. Only when children explicitly stated that they were just guessing, we included them in “Don’t know” category. The relationship between verbal explanations, age and condition was explored using chi-square tests.

### Results

#### Training

All children except for one 5 and three 3-year-olds passed the transparent training within 5 consecutive trials. Two of these children needed 6 and the other two needed 8 trials to complete the transparent training.

#### Test

The full model comprising of the interaction of age and condition, sex and trial number as fixed effects fit the data better than the null model which lacked these fixed effects [χ^2^(9) = 30.91, *p* < 0.001]. We found that there was a significant condition and age interaction [χ^2^(3) = 8.71, *p* < 0.05] and a significant effect of trial number [χ^2^(1) = 6.99, *p* < 0.01]. There was no effect of sex [χ^2^(1) = 3.17, *p* = 0.075] (Supplementary Table S1).

**FIGURE 2:**
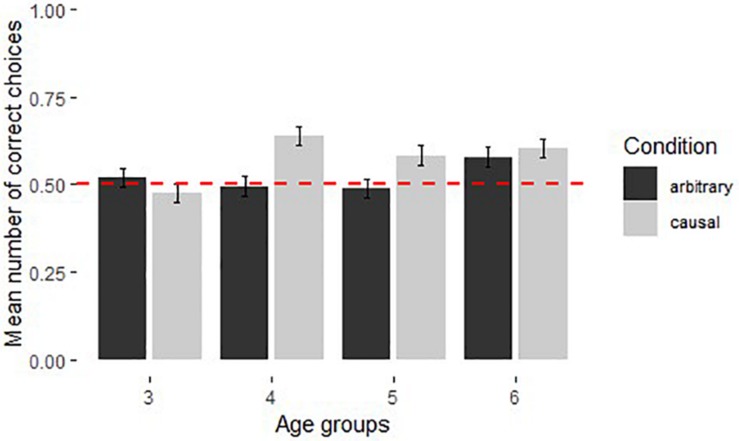
Performance of children in the causal and arbitrary conditions in Experiment 1 (*N* = 129, see Table 1 for age group information and means). Dotted line shows chance level performance (*p* = 0.05), error bars represent *SE*.

Comparisons of children’s performance in different conditions across age groups showed that there was no significant difference between performance in the causal and arbitrary conditions for 3- and 6-year olds (Mann–Whitney *U*-Test for 3-year-olds: *U* = 93, *N*_causal_ = 16, *N*_arbitrary_ = 16, *p* = 0.348; 6-year-olds: *U* = 133, *N*_causal_ = 17, *N*_arbitrary_ = 16, *p* = 0.921). Three-year-olds performed at chance level in both causal (Median: 0.45, Wilcoxon signed-ranks test: *T^+^* = 72, *N* = 15, *p* = 0.513) and arbitrary conditions (Median: 0.5, *T^+^* = 40, *N* = 11, *p* = 0.562); 6-year-olds were above chance in both causal (Median: 0.6, *T^+^* = 127, *N* = 17, *p* < 0.05) and arbitrary conditions (Median: 0.6, *T^+^* = 92.5, *N* = 14, *p* < 0.01). Four-year-olds performed significantly better (*U* = 64.5, *N*_causal_ = 16, *N*_arbitrary_ = 16, *p* < 0.05) and above chance levels in causal condition (Median: 0.62, *T^+^* = 98.5, *N* = 14, *p* < 0.01) as opposed to chance level performance in arbitrary condition (Median: 0.5, *T^+^* = 39, *N* = 12, *p* = 1). Five-year-olds showed a similar trend for better performance compared to chance in the causal condition (Median: 0.55, *T^+^* = 61, *N* = 12, *p* = 0.08) than in the arbitrary condition (Median: 0.48, *T^+^* = 66, *N* = 15, *p* = 0.751); however this difference was not significant (*U* = 93, *N*_causal_ = 16, *N*_arbitrary_ = 16, *p* = 0.191). Figure 2 shows the average performance of each age group in causal and arbitrary conditions. An effect of learning as evidenced by the significant effect of trial number on performance was found. This was expected given that subjects had no way of solving the task in their first trial.

There was no significant relationship between condition and side-bias [χ^2^(1) = 0.73, *p* = 0.39], however, there was a significant relationship between age and side bias [χ^2^(3) = 16.77, *p* < 0.001]. Three-year-olds were more likely to be side biased than other age groups.

#### Open Ended Question

Table 2 summarizes the percentages of children’s responses to the question “How did you decide which box to choose?” in each age group across two conditions. For a more robust analysis using chi-square, “no explanation” and “don’t know” categories; and “non-causal strategies” and “wrong causal explanations” categories were lumped to result in three explanation categories in total: “no idea,” “wrong idea,” and “correct explanation.” According to the chi-square analysis there was not a significant relationship between 3-year-old children’s explanations and the condition they were in [χ^2^(1) = 0.36, *p* = 0.55]. In both conditions, a high percentage of 3-year-olds had “no idea” about how to find the ball, a minority gave a wrong explanation and there were no children who could provide the correct explanation. There was a significant relationship between 4-year-olds’ explanations and condition [χ^2^(2) = 6.95, *p* < 0.05]. Although the majority of 4-year-olds were in the “no idea” category in both conditions, 33.3% could provide the correct explanation in the causal condition whereas none did in the arbitrary condition. Interestingly, there was a higher percentage of children in the arbitrary condition who gave a “wrong idea” explanation compared to those in the causal condition. The relationship between explanations and condition were marginally significant for 5-year-olds [χ^2^(2) = 5.73, *p* = 0.057]. “No idea” responses were comparable in both conditions, however, there were more 5-year-olds in the arbitrary condition who referred to wrong explanations than in causal condition and there were more children in causal condition that referred to the “correct explanation” than in the arbitrary condition. There was a significant relationship between 6-year-olds’ explanations and the condition they were in [χ^2^(2) = 6.51, *p* < 0.05]. The pattern was similar to 5-year-olds. More children referred to wrong explanations in the arbitrary condition compared to the causal condition and more 6-year-olds in the causal condition came up with an explanation based on different sounds than in the arbitrary condition.

**TABLE 2:** Percentage of children who gave the following explanations in response to the question “How did you know where the ball was?” in Experiment 1 (*N* = 114).

	Causal condition				Arbitrary condition			
Explanations	3 yo	4 yo	5 yo	6 yo	3 yo	4 yo	5 yo	6 yo
No idea	90%	58.3%	43%	31%	81%	67%	33%	34%
Wrong idea	10%	8.3%	21%	25%	19%	33%	60%	41%
Correct explanation	0%	33.3%	36%	44%	0%	0%	7%	25%

Finally we explored whether children’s reports matched with their performance. The performance of the 16 children who referred to different sounds/materials in the causal condition was compared with the performance of an age-matched group in the causal condition who gave other explanations. The model comprising of the fixed effects of explanations (correct/incorrect), trial number and sex fit the data better than the null model without the fixed effects [χ^2^(3) = 29.47, *p* < 0.001] (Supplementary Table S2). There was a significant effect of explanation type on children’s performance [χ^2^(1) = 24.90, *p* < 0.001]. Children who gave the correct explanations performed better than their peers who gave incorrect explanations [Mean difference = 0.25, 95% CI [0.15, 0.36], *t*(15) = 5.24, *p* < 0.001]. Moreover, children who gave correct explanations performed above chance levels [*M* = 0.77, 95% CI [0.69, 0.85], *t*(15) = 7.17, *p* < 0.001], whereas those who gave incorrect explanations were at chance [*M* = 0.52, 95% CI [0.47, 0.56], *t*(15) = 0.76, *p* = 0.46].

### Discussion

When the sound cues were consistent with a causal structure, by 4-years of age children used the discriminatory sound cue to locate the ball, whereas 3-year-olds failed. When the cues were not consistent with a causal structure, 4–5-year-olds did not use these same sounds to find the ball; and performed worse than they did in the causal condition. This difference was significant for 4-year-olds but not for 5-year-olds. These results suggested that children went beyond the immediately available cues to imagine their likely unseen causes. The explanations children provided about how they found the ball matched the results of the main task. More children referred to different sounds/materials when there was a plausible causal structure than when the relation was arbitrary. In addition, the children who referred to different sounds outperformed their peers who gave different explanations for their choice.

However, one could argue that the temporal proximity between the distinct sound cue (metal/wood) and the outcome (choice of one box) was smaller in the causal condition: when the order of events was “falling” (filler) sound, metal/wood sound, choice, than in the arbitrary condition, when the order was metal/wood sound, filler sound, and then choice. And since associations are more easily formed between temporally proximate events (Barnet et al., 1991; Miller and Barnet, 1993), and even brief delays have been shown to result in a reduction of causality judgments (Michotte, 1963; Shanks et al., 1989), these could explain the better performance in the causal condition compared to the arbitrary condition. In Experiment 2 we tested this alternative explanation.

Six-year-olds performed equally well in both conditions. Their successful performance in the arbitrary condition might have resulted from the ability to treat arbitrary cues as symbols to solve a problem (DeLoache, 2004; Seed et al., 2011; Mayer et al., 2014). On the other hand, 3-year-olds did not pass either condition in this study, they were unable to provide a verbal explanation about how they found the ball and were more likely to be side-biased.

One possibility for the failure of 3-year-olds could be that unlike older children, they cannot, or do not spontaneously, imagine unseen causes. However, other explanations are possible too, such as the necessity to remember the cues which, being auditory, are transitory, and map them to one of the two boxes which do not look to be made of the materials evoked by the sounds. In Experiment 4 we simplified the task by using boxes that were visibly made of metal and wood, to examine whether or not this task would be easier. In Experiment 3, we tested chimpanzees, and planned to titrate the level of difficulty based on our initial results with the task described above.

## Experiment 2: Follow-Up With Arbitrary Sounds

In this experiment, we tested whether better performance in the causal condition as opposed to the arbitrary condition in Experiment 1 could be due to temporal proximity of the sound cues and the outcome. Children were asked to locate a sticker in one of the two boxes based on recorded sounds which were similar either to the causal (filler, wood/metal) or the arbitrary order (wood/metal, filler) of the Experiment 1. Would children perform better when the discriminatory cue was more proximate to the choice, than when it was followed by a filler sound? If this was the case, then it would raise concerns that the differences between the causal and arbitrary conditions in Experiment 1 could be due to temporal proximity rather than causal plausibility. However, if children detected the causal structure, we did not expect to find differences between conditions when all cues, regardless of the order, were arbitrarily related to the outcome.

### Methods

#### Participants

A new group of 40 4–5-year-old children were tested. Half of them participated in the *filler discriminatory* condition and the other half participated in the *discriminatory, filler* condition. Age and sex were split roughly equally in the two conditions (Table 1). Two additional children that were tested were excluded from the study due to refusal to complete the task.

#### Materials

The yellow and gray boxes at the bottom of the channels in Experiment 1 were used. The boxes were covered with lids so that children could not see inside. A barrier (52 × 31 cm) concealed the hiding event. Two sounds that lasted about 1 second were recorded and played back to the children from the experimenter’s phone. The sounds were amplified with a speaker.

#### Procedure

##### Test

The experimenter introduced the task saying; “In this game, I will hide a sticker in one of these boxes. You won’t see where it goes but there is a way to find the sticker in the correct box. I will give you the clue using my phone! If you point to the correct box, you will win the sticker. Ready?” The experimenter then hid the sticker behind the barrier. Upon removing the barrier, she said “Now, pay attention!” and played the recorded sound from her phone. In the *filler, discriminatory* condition, the children heard the filler sound followed by a metal/wood sound at the end and in the *discriminatory, filler* condition, they heard the metal/wood sound followed by the filler sound. The experimenter asked “Where do you think is the sticker?” and the child pointed to or opened the box that she/he thought the sticker was in. Once the child made a choice, the other box was also opened to show the content.

In total children got 20 trials which lasted about 10 min. The side of the boxes was randomized across subjects. The sticker was placed in each box ten times in a random fashion but never in the same box more than twice in succession.

##### Open ended question

At the end of the task, the experimenter asked; “How did you decide which box to choose?” If children did not reply, the experimenter elaborated “Sometimes the ball was in the gray one and sometimes in the yellow one. How did you know where the ball was?” A second examiner categorized children’s answers into these five different types of explanations, *Kappa* = 0.92 [95% CI, 0.85, 0.96], *p* < 0.001.

#### Scoring and Analysis

A second examiner coded 20% of the videos for reliability, *Kappa* = 0.98 [95% CI, 0.97, 0.98], *p* < 0.001. The full model consisted of condition, trial number and sex as fixed effects; ID and the side of the boxes as random effects. We also included random slopes of trial number within ID, but left out the correlation parameters between random intercepts and random slopes terms (Schielzeth and Forstmeier, 2009; Barr et al., 2013). This full model was compared to a null model which included only the random effects using a likelihood ratio test.

The model was stable, overdispersion was not an issue with regards to the full model (dispersion parameter: 0.86) and there was no multicollinearity. We used one-sample *t*-tests to examine performance different from chance level (*p* = 0.05) in the two conditions. Finally, the relationship between verbal explanations and condition was explored using chi-square tests.

### Results

#### Test

**FIGURE 3:**
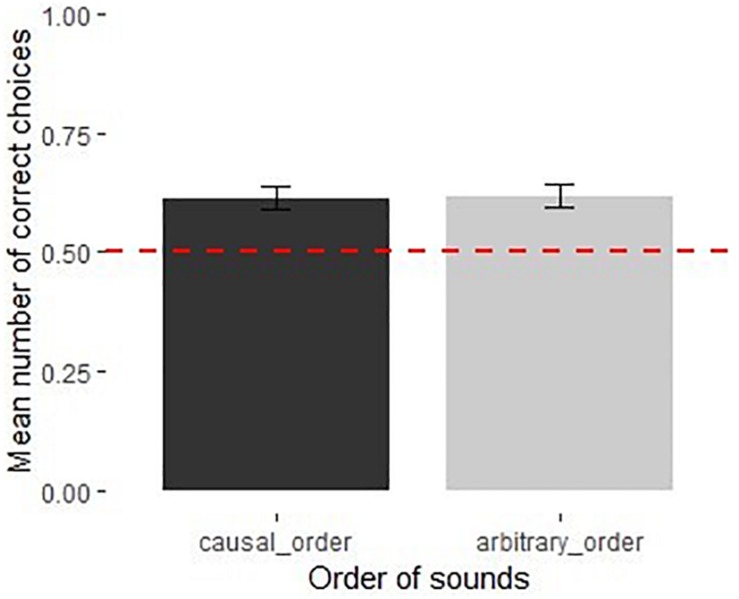
Performance of children in the causal and arbitrary recorded sound orders in Experiment 2 (*N* = 40). Dotted line shows chance level performance (*p* = 0.05), error bars represent *SE*.

The full model was not significantly different from the null model [χ^2^(3) = 0.38, *p* = 0.945]. None of the predictors had a significant influence on performance (Supplementary Table S3). Children performed above chance in both the causal [*M* = 0.61, 95% CI [0.52, 0.71], *t*(19) = 2.48, *p* < 0.05] and the arbitrary sound orders [*M* = 0.62, 95% CI [0.53, 0.70], *t*(19) = 2.87, *p* < 0.01] (Figure 3).

#### Open Ended Question

There were 2 missing data points so the analysis was conducted on data from 38 children. There was not a significant relationship between children’s explanations and the condition they were in [χ^2^(2) = 3.03, *p* = 0.22]. Overall, there were 13 children who were in the “no idea” group; 14 children in “wrong explanations” and 11 children who gave the correct explanation. Only the children who were in the correct explanation group performed above chance level [*t*(10) = 7.24, *p* < 0.001].

### Discussion

When the sound cues to locate the reward was completely arbitrary, the order children heard them did not influence their performance and there was no relationship between their verbal explanations and the condition they were in. Therefore, better performance in the causal than the arbitrary condition in Experiment 1 could not simply be explained based on the temporal proximity of the sound cue and the outcome as the associative accounts would suggest. Indeed, Buehner and May (2002, 2003) have also challenged the necessity of temporal proximity for causal judgments by showing that it was the knowledge of the causal structure that influenced participants’ judgments.

Overall, this experiment provided further evidence to support our interpretation that by 4-years of age children were able to use indirect evidence to detect unseen causes based on data alone. In Experiment 3 we explore chimpanzees’ abilities to detect unseen causes.

## Experiment 3: Chimpanzees

The experiment with chimpanzees consisted of two phases. In the first phase, we planned to test 6 subjects in the causal condition as described in Experiment 1. If subjects passed the causal condition, in the second phase, we planned to test a further 6 chimpanzees on the arbitrary condition. However, if they did not pass the causal condition, in the second phase we planned to simplify the task by replacing the yellow and gray boxes at the bottom with metal and wooden boxes (familiar boxes). With this manipulation the subjects would receive additional visual feedback with the conspicuously metal and wooden boxes that could help them match the sounds and materials more easily.

### Methods

#### Participants

Chimpanzees housed at the Wolfgang Köhler Primate Research Center, Leipzig Zoo (Germany), were selected by convenience sampling. Six chimpanzees participated in the first phase: causal condition with unfamiliar boxes. Because none of these individuals passed the task at above chance levels, in the second phase, 3 of these experienced chimpanzees and 3 additional naïve subjects were assigned to the “Familiar boxes, causal condition” and the other 3 experienced and 3 additional naïve subjects were assigned to the “Familiar boxes, arbitrary condition.” One subject in the “familiar boxes arbitrary condition” stopped approaching the mesh for testing after a few sessions, so she was dropped from the study, leaving 11 subjects in total who participated in the second phase (Table 3). Subjects lived in two groups of 6 and 19 individuals and had access to indoor and outdoor enclosures. They were tested individually in their sleeping rooms and were not deprived of food or water at any time. Testing days were consecutive as much as possible. If a subject did not choose to participate, testing for this individual was canceled for that day. Research was conducted in accordance with the regulations of the University of St Andrews’ Animal Welfare and Ethics Committee (AWEC), Max Planck Institute for Evolutionary Anthropology and Zoo Leipzig.

**TABLE 3:** The name, age, sex, rearing history and information about experiment participation of chimpanzees (*N* = 11).

Name	Age	Sex	Rearing history	Participation (condition)
Hope	26	f	Nursery	Unfamiliar (causal), familiar (causal)
Kofi	11	m	Mother	Unfamiliar (causal), familiar (causal)
Fraukje	41	f	Nursery	Unfamiliar (causal), familiar (causal)
Bangolo	7	m	Mother	Familiar (causal)
Sandra	24	f	Mother	Familiar (causal)
Lobo	13	m	Mother	Familiar (causal)
Tai	14	f	Mother	Unfamiliar (causal), familiar (arbitrary)
Dorien	36	f	Nursery	Unfamiliar (causal), familiar (arbitrary)
Riet	39	f	Nursery	Unfamiliar (causal), familiar (arbitrary)
Lome	15	m	Mother	Familiar (Arbitrary)
Frodo	23	m	Mother	Familiar (arbitrary)

#### Materials

##### Transparent training and opaque testing boxes

Exact replicas of the apparatuses described in Experiment 1 were used to test chimpanzees in the unfamiliar boxes causal condition (Figure 1). The apparatus was placed on a sliding table (78 × 38 cm) which was fixed to the sides of the mesh panel (78 × 55 cm). A second opaque screen was placed behind the mesh panel to block the view of the subject in between trials.

**FIGURE 4:**
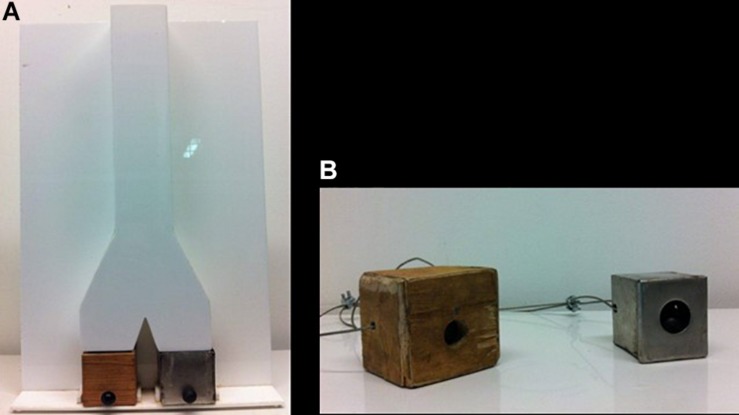
Testing box used in familiar boxes (wooden and metal) conditions **(A)** and sound-making training boxes **(B)** in Experiment 3.

In the second phase with familiar boxes, the yellow and gray boxes were replaced with boxes of the same size made of wood and metal (Figure 4).

##### Sound making training boxes

Chimpanzees in Leipzig Zoo had objects made of different materials in their outdoor and indoor enclosures (i.e., automatic metal feeders, tree logs, plastic buckets) and occasionally may hear the noises they make when they are hit/dropped. However, in comparison to children we assumed their exposure to metal and wooden materials would be limited. Therefore, we prepared two sound-making training boxes: the “metal box” (6 × 5.5 × 6 cm) made from stainless steel and the “wooden box” (8 × 7 × 7 cm) made from ply-wood. In both boxes, there was a thermoplastic ball (1.30 cm in diameter); and there was a hole (1.25 cm in diameter) on one side of the box. The boxes also contained peanuts which could be shaken free, an action which caused the ball to rattle inside the box and make sounds. The boxes were passed to the chimpanzees through a movable feeder that was adjacent to the mesh panel they were tested. A steel wire passing through each sound-making box secured them to the steel feeder. Therefore, the subjects could play with the boxes but could not take them away (Figure 4).

#### Procedure

##### Training phase

All subjects completed the transparent training phase before moving on to the testing. The experimenter placed the transparent apparatus on the sliding table, put a food reward (dates, peanuts based on subjects’ preference) in the ball and when the subject was sitting in front of the mesh, dropped the ball from the top opening. The subjects were highly motivated to find the high value food rewards, and were familiar with the experimental setup where they tried to locate rewards in cups/boxes/apparatuses. When the experimenter pushed the sliding table toward the mesh, the subjects could point to one of the boxes at the bottom. If the subject chose correctly, the experimenter gave the reward to the subject and took out the other box to show that it was empty. If the subject pointed to the wrong box, the experimenter first showed the empty box and then showed the content of the other box and put the food reward back into the bucket. If the subject pointed to an irrelevant location or the choice was ambiguous, the experimenter pulled the sliding table back, tapped on both boxes at the same time and pushed the table forward again. When a trial was over, the opaque screen was put behind the mesh. Chimpanzees received 10 trials per session and training continued until the subjects selected the correct side 16 out of 20 trials or more (a binomial test was run to calculate *p*-value, *p* = 0.004).

Once a subject passed the transparent training the subjects also received the sound-making boxes training. The experimenter put shelled peanuts in full view of the subject into one of the boxes and passed it to the subject using the steel feeder. When the subject shook the boxes, the ball hit the walls of the box making metal/wooden sounds and the peanuts came out through the hole. Once the subject was done with one box, the experimenter replaced it with the other box. Half of the subjects got the metal box first and the wooden second and the other half did the reverse order. They got sound-making boxes training at the beginning of each testing session.

##### Test phase

The *unfamiliar boxes causal condition* was the same as described above in Experiment 1. Chimpanzees got 10 trials per session and testing ended when a subject selected the correct side 16 out of 20 times or more or until 10 sessions were completed. The side of the yellow/gray boxes at the end of the channels were randomized across subjects. The ball was placed in each box 5 times in a random fashion but never in the same box more than twice in succession.

The procedure for the *familiar boxes conditions* with wooden and metal boxes at the bottom were the same as the unfamiliar boxes.

#### Scoring and Analysis

A second examiner coded 20% of the videos for reliability, *Kappa* = 0.81 [95% CI, 0.75, 0.87], *p* < 0.001. In the *unfamiliar boxes causal condition* the full model comprised of age, sex, session and trial numbers as fixed effects and ID and the side of the boxes as random effects. We included random slopes of trial and session numbers within ID, but left out the correlation parameters between random intercepts and random slopes terms. The full and null model comparison was done using a likelihood ratio test. In order to explore performance in this condition against chance level (*p* = 0.05) we used a one-sample *t*-test. In the *familiar boxes conditions*, same analyses methods were used with the addition of condition (causal/arbitrary) and experience (experienced/naïve) to fixed effects.

Both models for unfamiliar and familiar boxes were stable and there were no issues with regards to overdispersion (*dispersion parameter: 1.01* for both*)*, however, multicollinearity was an issue for the predictors, age and sex. Therefore, sex was removed from the models.

### Results

#### Unfamiliar Materials

##### Transparent training

All chimpanzees except for one reached the criterion in the transparent training within two sessions which was the minimum amount. This subject needed an extra session to reach the criterion.

##### Testing

**FIGURE 5:**
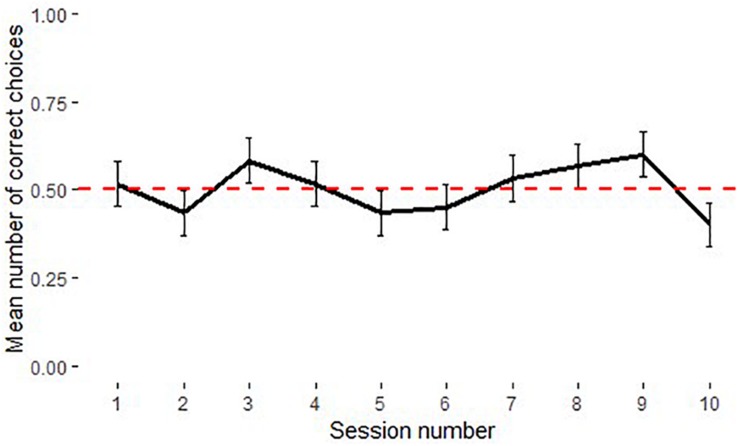
Performance of chimpanzees in the unfamiliar boxes causal condition across sessions in Experiment 3 Phase 1 (*N* = 6). Dotted line shows chance level performance (*p* = 0.05), error bars represent *SE*.

None of the subjects reached the criterion in the unfamiliar boxes causal condition; therefore, all subjects received 10 sessions (see Figure 5). The full model was not significantly different from the null model [likelihood ratio test: χ^2^(3) = 1.43, *p* = 0.698] (Supplementary Table S4). Furthermore, they were at chance level overall as a group [*M* = 0.50, 95% CI [0.45, 0.56], *t*(5) = 0.15, *p* = 0.885]. All individuals except for one were side biased.

Since none of the subjects in unfamiliar boxes condition passed the task, we moved on to the familiar boxes.

#### Familiar Materials

##### Transparent training

Five naïve subjects got the transparent training before moving on to the testing sessions. They reached the criterion within two sessions.

##### Testing

**FIGURE 6:**
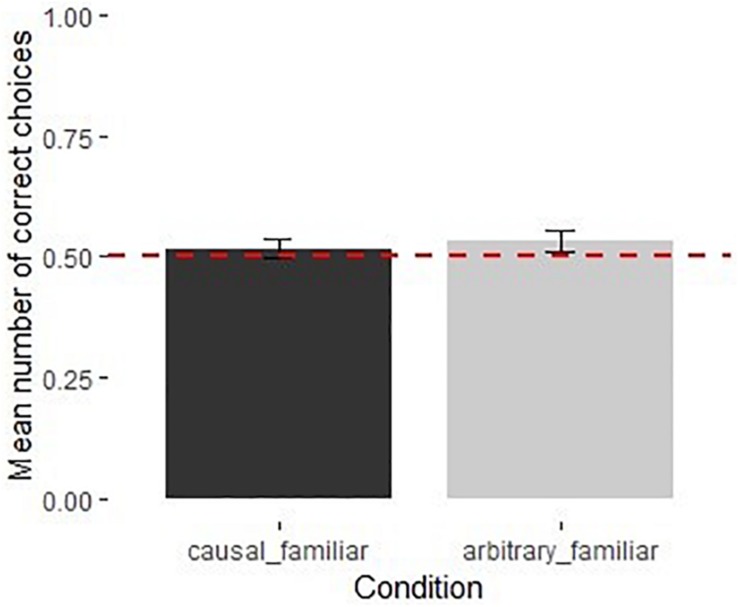
Performance of chimpanzees in the familiar boxes causal and arbitrary conditions in Experiment 3 Phase 2 (*N* = 11). Dotted line shows chance level performance (*p* = 0.05), error bars represent *SE*.

The model including condition, experience level, age, session and trial numbers was not significantly different from the null model [χ^2^(5) = 2.74, *p* = 0.741]. There was no significant difference between performances in the causal and arbitrary conditions nor between the performances of experienced and naïve individuals (Supplementary Table S5). Subjects in both conditions performed at chance level; familiar boxes causal [*M* = 0.52, 95% CI [0.46, 0.57], *t*(5) = 0.76, *p* = 0.480] and familiar boxes arbitrary [*M* = 0.53, 95% CI [0.50, 0.56], *t*(4) = 2.67, *p* = 0.06] (Figure 6). One subject reached the criterion in the causal familiar condition in the last session (*M* = 0.62, *SD* = 0.16); whereas none of the subjects in the arbitrary condition passed the task. All individuals except for one in the familiar boxes arbitrary condition were side biased. There was no significant relationship between condition and side bias [χ^2^(1) = 1.32, *p* = 0.251].

### Discussion

Chimpanzees were at chance level in both the causal and arbitrary conditions and there were no significant differences between them. Negative results are difficult to interpret, and while the results, for chimpanzees as for 3-year-olds, could speak to limitations in spontaneously imagining an unseen cause, there are other explanations that could account for their failure. For example, the requirement to integrate knowledge about how the channels worked (i.e., the ball can land in any one of the boxes randomly) with the sounds of different materials could have been challenging. In a recent study, chimpanzees did not spontaneously cover the two exits of a similar forked chute, suggesting that this kind of event might be difficult for them to anticipate (Suddendorf et al., 2017). Therefore, in Experiment 4, we simplified the task further by removing the channels completely, and simply requiring subjects to infer where the ball was based on the sound of one of the boxes being shaken.

## Experiment 4: Shaken Boxes

We aimed to see if children and chimpanzees could infer the location of a food reward in one of two boxes (made of wood and metal) based on the different sounds made when a ball was shaken in one of the boxes behind a barrier. We predicted that 4- and 5-year-olds would be able to imagine the cause of the sound and choose the box made of the corresponding material, since such an ability would be a pre-requisite for their success in Experiment 1. Given that 3-year-olds and chimpanzees have been shown to infer the location of a reward based on the presence or absence of a sound cue in previous research (Call, 2004; Hill et al., 2012), we could predict that they would do so here, if they were able to match the sound made by the different materials to the appearance of the boxes. We therefore predicted that they would perform better than they did in Experiments 1 and 3.

### Methods

#### Participants

Eleven chimpanzees (same as in Experiment 3) and a new group of 48 3–5-year-old children (16 in each age group) participated in this study (Table 1). Four additional children that were tested were excluded from the study due to parental interference (2) and refusal to complete the task (2).

#### Materials

The metal and wooden boxes from Experiment 3 were used. The boxes were covered with lids to block subjects’ view. The ball was made from thermoplastic (1.30 cm in diameter). A barrier was used to occlude the hiding and shaking events.

#### Procedure

##### Children

The experimenter placed the boxes (approximately 15 cm apart from each other) and the ball on the table and introduced the task to the children: “In this game I have these two boxes. Now I will put a sticker in the ball and I will hide the ball in one of them. If you can find the ball, you will win the sticker!” Then the experimenter put the barrier in between and hid the ball in a box and shook it for approximately 5 s; and said “Here is a clue!” Children could see the arms of the experimenter but not the box being shaken. Then the experimenter placed the boxes in their original positions, removed the barrier and asked “Which box do you want to open?” Children received ten trials. The location of the boxes were counterbalanced.

At the end of the task, the experimenter asked children how they found the ball as in Experiment 1.

##### Chimpanzees

The procedure was the same for chimpanzees as in children apart from verbal instructions. Chimpanzees received 10 trials per session and testing continued until the subjects selected the correct side 16 out of 20 trials or more or until 10 sessions were completed.

#### Scoring and Analysis

A second examiner coded 20% of the videos for reliability, *Kappa* = 1.00, *p* < 0.001 for both children and chimpanzees. The full model based on the child data comprised of age, sex, and trial number as fixed effects. The full model of the chimpanzee data comprised of age, sex, session and trial numbers as fixed effects. For both models, ID and the side of the box were the random effects. We included random slopes of trial (and session numbers for chimpanzees) within ID but left out the correlation parameters between random intercepts and random slopes terms. The full and null model comparisons were done using a likelihood ratio test. In order to explore performance in this experiment against chance level (*p* = 0.05) we used one-sample *t*-tests.

Both models for children and chimpanzees were stable and there were no issues with regards to overdispersion (*dispersion parameter for children: 0.71; for chimpanzees: 0.99).* There was no multicollinearity issue for child data; however, age and sex predictors resulted in collinearity in chimpanzee data. Therefore, sex was dropped from the model.

### Results

#### Children

**FIGURE 7:**
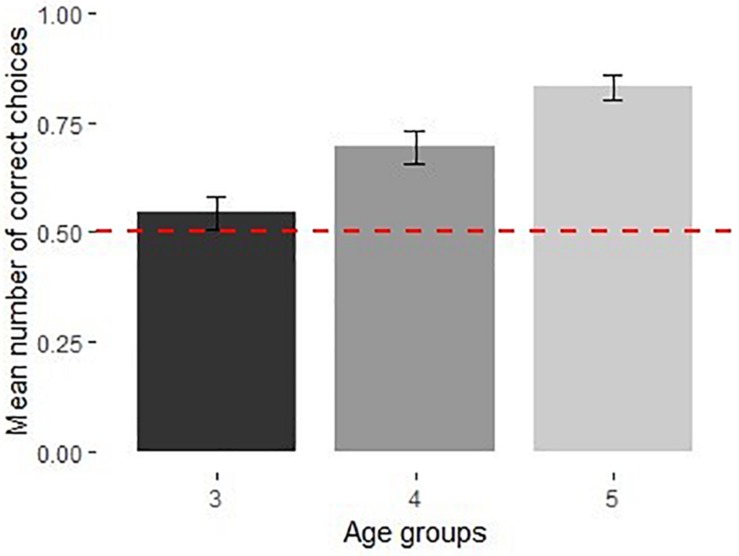
Performance of 3–5-year-olds in shaken boxes in Experiment 4 (*N* = 48). Dotted line shows chance level performance (*p* = 0.05), error bars represent *SE*.

The full model was significantly different from the null model [χ^2^(4) = 16.62, *p* < 0.01]. There was a significant effect of age, [χ^2^(2) = 15.26, *p* < 0.001], no effect of sex [χ^2^(1) = 0.142, *p* = 0.706] and no effect of trial number [χ^2^(1) = 1.21, *p* = 0.271] (Supplementary Table S6). The pairwise comparisons between age groups showed that there was a significant difference between the performances of 3- and 5-year-olds (GLMM, user-defined contrasts, z = 3.87, *p* < 0.001); no differences between 3- and 4-year-olds (*z* = 1.89, *p* = 0.140) nor between 4 and 5-year-olds (*z* = 2.17, *p* = 0.08) (see Table 1 for means). Three-year-olds performed at chance [*M* = 0.54, 95% CI [0.44, 0.64], *t*(15) = 0.94, *p* = 0.362], 4- and 5-year-olds were significantly above chance; [*M* = 0.69, 95% CI [0.58, 0.81], *t*(15) = 3.61, *p* < 0.01] and [*M* = 0.83, 95% CI [0.71, 0.95], *t*(15) = 5.91, *p* < 0.001] respectively (Figure 7).

Table 4 summarizes the percentage of children in each age group based on their responses to the open-ended question. When the replies were lumped into three explanation categories as in Experiment 1, there was a significant relationship between age groups and explanations [χ^2^(4) = 20.46, *p* < 0.001]. The majority of the 3-year-olds were in the “no idea” category (88%) and only 1 (6%) gave the correct explanation. Among 4-year-olds, 40% were in the “no idea,” 27% were in the “wrong idea” categories but 33% of them gave correct explanations. Among 5-year-olds only 31% were in the no or wrong idea categories and the majority (60%) were able to provide the correct explanation.

**TABLE 4:** Percentage of children who gave the following explanations in response to the question “How did you know where the ball was?” in Experiment 4 (*N* = 48).

	Shaken boxes		
Explanations	3 yo	4 yo	5 yo
No explanation	88%	40%	13%
Wrong idea	6%	27%	19%
Correct explanation	6%	33%	69%

In order to examine whether children’s reports matched with their performance, the performance of the 11 children who referred to different sounds/materials was compared with the performance of an age-matched group who gave other explanations. Those who referred to different sounds/materials performed significantly better (*M* = 0.93, *SE* = 0.04) than those who gave other explanations [*M* = 0.62, *SE* = 0.06), *t*(20) = 4.26, *p* < 0.001].

#### Chimpanzees

**FIGURE 8:**
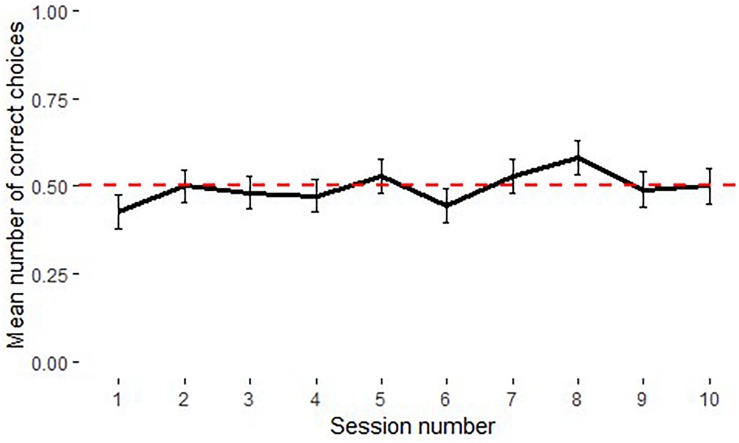
Performance of chimpanzees in shaken boxes across sessions in Experiment 4 (*N* = 11). Dotted line shows chance level performance (*p* = 0.05), error bars represent *SE*.

The full model for the chimpanzee data did not differ from the null model [χ^2^(3) = 2.41, *p* = 0.492] (Supplementary Table S7). Chimpanzees performed at chance level [*M* = 0.50, 95% CI [0.46, 0.53], *t*(10) = -0.20, *p* = 0.844] (Figure 8). However, one subject passed the shaken boxes condition in the 8th session. All but three individuals were side biased.

### Discussion

Three-year-olds and chimpanzees could not infer the location of the ball based on auditory evidence about the material of a shaken box. In line with the previous findings from Experiment 1, we found that 4- and 5-year-olds performed significantly above chance level, corroborating the conclusion that by 4 years of age children are capable of reasoning about evidence to detect unseen causes in the absence of linguistic scaffolding.

In our last experiment we explored whether 3-year-olds’ performance would improve with the addition of causal language as suggested by previous literature (Bonawitz et al., 2010; Butler and Markman, 2012; Lane and Shafto, 2017). We used the shaken boxes paradigm but this time provided cues to the causal structure of the task verbally.

## Experiment 5: Follow Up With Causal Language

### Methods

#### Participants

A new group of 28 3-year-old children participated. There were equal numbers of boys and girls (Table 1). Three additional children that were tested were excluded from the study due to refusal to complete the task (2), and difficulties with language (1).

#### Materials

Same boxes were used as in Experiment 4.

#### Procedure

The procedure was the same as in Experiment 4 with the only exception of the question we asked children to locate the ball. Instead of “Which box do you want to open?” the experimenter asked “Which box did I shake?”

A second examiner categorized children’s answers into five different types of explanations, *Kappa* = 0.97 [95% CI, 0.94, 0.99], *p* < 0.001.

#### Scoring and Analysis

A second examiner coded 20% of the videos for reliability, *Kappa* = 1.00, *p* < 0.001. In order to see the influence of a causal question on 3-year-olds’ performance, we merged the data from Experiment 4 with the current data. Our model consisted of question type (non-causal as in Experiment 4/causal), trial number and sex as fixed effects; ID and the side of the boxes as random effects. We also included random slopes of trial number within ID, as well as the correlation parameters between random intercepts and random slopes terms (Schielzeth and Forstmeier, 2009; Barr et al., 2013). The full model was compared to a null model which included only the random effects using a likelihood ratio test.

The model was stable with regards to the predictors, there were no issues with regards to overdispersion (*dispersion parameter:* 0.85), nor multicollinearity.

We used one-sample *t*-test to examine whether children’s performance was significantly different from chance level (*p* = 0.05).

### Results

**FIGURE 9:**
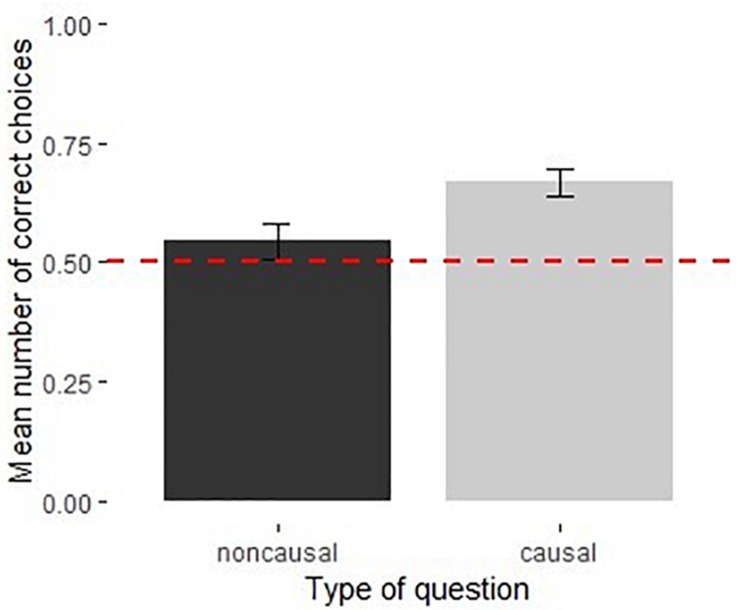
Performance of 3-year-olds in shaken boxes when they were asked a non-causal (*N* = 16, Experiment 4) vs. a causal question (*N* = 28, Experiment 5). Dotted line shows chance level performance (*p* = 0.05), error bars represent *SE*.

The full model was not significantly different from the null model [χ^2^(3) = 5.44, *p* = 0.142] (Supplementary Table S8). However, we found that 3-year-olds performed significantly above chance levels in the follow-up [*M* = 0.67, 95% CI [0.57, 0.76], *t*(27) = 3.60, *p* < 0.01] as opposed to their chance level performance in the absence of causal language (Figure 9).

The majority of the 3-year-olds were in the “no idea” category (71%) but 18% gave the correct explanation.

### Discussion

Three-year-olds performed significantly above chance levels when they were asked a question that hinted at the causal structure of the shaken boxes task as opposed to chance level performance in Experiment 4. Even though the majority still could not explain how they found the ball, more children than in Experiment 1 gave the correct explanation. These findings showed that 3-year-olds were able to distinguish the auditory stimuli, and the peripheral demands of remembering what they heard and matching the sound with the box were not too high. However, this experiment does not explain how exactly verbal framing of the task facilitated performance. One possibility is that, the causal question boosted their performance through highlighting the problem; hence, scaffolding their ability to make inferences. Another possibility is that, by asking a question like “Which box did I shake?” we simplified the task such that it reduced the need for children to seek a causal explanation for the sounds they heard.

## General Discussion

We presented 3–6-year-old children and chimpanzees with a novel, natural causation task where they needed to use indirect evidence (auditory cues) to locate a reward in the absence of either a directly-perceivable causation relationship, or a verbal instruction to look for the cause of an outcome. By 4-years of age, children were able to make causal inferences based on evidence alone. Importantly, they only did so in the causally-plausible condition in which the falling ball could have caused the different sounds, rather than when the sound cues preceded the dropping of the ball and therefore bear an arbitrary relationship to its final location. In Experiment 2, we corroborated these findings by eliminating a simpler explanation for better performance in the causal condition than in the arbitrary one. Six-year-olds performed equally well and above chance levels in both causal and arbitrary conditions. This was in line with previous evidence showing that they are able to use arbitrary cues as meaningful symbols to solve a problem, in addition to detecting a causal structure from data. On the other hand, 3-year-olds and chimpanzees failed the task (Experiment 1 and 3). In Experiment 4, when the task was simplified to inferring the location of a reward in a metal and a wooden box based on the sounds it made, the performance of younger children and chimpanzees did not improve. But when the task was framed using causal language, 3-year-olds performed above chance levels. We discuss these findings and their implications in turn.

In the absence of causal instruction, 4- and 5-year-old children were able to use indirect auditory cues to locate a reward when there was a plausible causal structure to the task. They performed worse when the cues were arbitrarily related to the location of the reward. Children’s explanations corroborated these findings: they referred to different sounds and materials in the causal condition more than the arbitrary condition and those who referred to different sounds outperformed their peers who gave other explanations.

Similar to the 4- and 5- year-olds, 6-year-olds passed the causal condition, but in contrast to the younger children they performed equally well in the arbitrary condition. This was as predicted based on similar findings with this age group in previous studies (DeLoache, 2004; Mayer et al., 2014). We suspect that older children solved the task because they interpreted the arbitrary cues as symbolic. DeLoache refers to this ability as holding dual representations: representing the symbol as an object/event by itself and also as a cue that stands for something else. In the arbitrary condition of our task, the metal and the wooden sounds that came before the ball was dropped had no causal relevance to the task, however, they could be treated as symbols that cued the child to which box the ball would be in since they were always predictive of the ball’s location. Holding dual representations is cognitively challenging since one has to ignore the fact that it is causally irrelevant given the task but it may point to some information that is useful in order to solve the problem. For this reason DeLoache (2004) argued that the use of symbolic knowledge emerges fairly late in development, especially in the absence of verbal scaffolding.

Three-year-old children and chimpanzees did not discriminate between the conditions, did not pass either of them and were more likely to be side biased. This could reflect a “true negative”: perhaps 3-year-olds and chimpanzees do not spontaneously make inferences about unseen causes when dealing with this kind of indirect evidence. Previous research has shown causal reasoning abilities in 3-year-olds in the context of direct causal relations and/or with explicit verbal scaffolding (Gopnik et al., 2001; Sobel et al., 2004; Bonawitz et al., 2010), but when they were presented with indirect causal structures such as a block activating a machine at a distance (Kushnir and Gopnik, 2007) or a task required them to represent prior knowledge to solve a problem (Sobel et al., 2004), 3-year-olds performed at chance level. Although suggestive of inferential reasoning abilities, the studies conducted with chimpanzees (Call, 2004; Hanus and Call, 2008) were criticized for not eliminating simpler associative explanations (Penn and Povinelli, 2007) or simplifying the task largely by using food itself as a cue (Völter and Call, 2014). We found no evidence to suggest that chimpanzees imagined or reasoned about the unseen causes involved in this study based on evidence alone. At face value, these findings may support a number of past claims in the literature (Penn and Povinelli, 2007): that non-human primates do not engage in inferential causal reasoning.

However, as with many negative findings, interpreting these results is not straightforward. One explanation for the failure of chimpanzees could be that the initial training we implemented were not sufficient to build the necessary knowledge for solving the problem. We used the transparent channels and sound-making boxes training separately to provide them with the required information for solving the test. One could argue that, integrating these two pieces of information might have been challenging. An alternative would be to incorporate these two together (i.e., tracking the movement of an object based on two different sounds in a forked apparatus). However, providing animals with training that is highly similar to the test phase makes it difficult to rule out associative explanations for success. In addition, chimpanzees’ failure in Experiment 4 with shaken boxes despite the repeated experience with wood and metal sound-making boxes makes us more confident that the lack of prior experience was not the limiting factor.

Another explanation for the failure of chimpanzees could be limitations in executive function, specifically attention and working memory, which could mask or constrain their ability to reason. All of our tasks required subjects to integrate prior knowledge about object-object interactions with evidence to make inferences, and to keep track of transient auditory cues and match them with two different boxes. Although there is evidence that chimpanzees are sensitive to different sounds (Slocombe et al., 2009), capable of cross-modal matching (Davenport et al., 1973; Hashiya and Kojima, 2001), and inferring rewards based on auditory information (Call, 2004), performing all of these tasks at once may have overloaded their attention and working memory capacities. In support of this argument, Call (2007) found that; great apes failed to integrate information about the quality and the size of the reward when they were trying to locate a desirable piece of food under one of the two slanted boards although they were capable of doing these two tasks separately.

On the other hand, 3-year-olds’ failure to succeed cannot easily be explained based on limitations in executive functions. It is true that our first experiment might have been challenging for young preschoolers, as it relied on integrating knowledge about channels and the sound of different materials that were not visible. The cognitive control abilities such as attention shifting and working memory undergo significant changes between the ages three and four (Frye et al., 1995; Zelazo and Muller, 2002) and this may influence 3-year-olds’ performance when dealing with tasks where they need to keep track of multiple pieces of information. Previous research has shown how task difficulty may hamper performance of this age group (Hill et al., 2012). However, when we reduced the task difficulty largely with the removal of the channels and use of conspicuously metal and wooden boxes, it did not help 3-year-olds. On the other hand, they were able to solve the exact same task when causal language was involved, showing that the demands on executive functions were not too high. They were able to distinguish the two sounds, remember them at the time of choice and match them with the correct box. This brings us to the function of causal language.

How exactly verbal framing facilitates 3-year-olds’ performance remains unclear. One possibility is that 3-year-olds fundamentally have the same cognitive machinery as 4- and 5-year-olds. Causal instructions/questions only highlight the problem among other irrelevant stimuli. In Experiment 5, as opposed to Experiment 4, children no longer needed to imagine that the boxes were shaken and this was causing the sounds they heard. This information was provided by the experimenter and hence they only needed to focus on the evidence to detect the true cause without imagining unseen actions or object-object interactions. Their true capacity was brought out by this verbal scaffold. The second possibility is that, with such causal questions the task is no longer measuring causal reasoning. From this point of view, the children were not required to make spontaneous inferences about evidence anymore but were asked to match a sound with the correct box. Which of these interpretations better explain the difference we find between Experiment 4 and 5 is an open and an interesting question that requires further research.

Overall, children’s explanations about how they found the ball were in line with their problem-solving performance. First, there were more children in the causal condition than in the arbitrary one who said they found the ball based on different sounds it made in different boxes. Second, these children’s explanations aligned with their performance: they performed better than those who referred to other explanations. Third, most of the 3-year-olds who performed at chance levels in both conditions either could not provide a verbal explanation or said they did not know how to find the ball implying that they found the task challenging. In addition when 3-year-olds’ performance improved in the last experiment with causal language, this was reflected in their verbal explanations too: there were more children who gave correct explanations compared to Experiment 4. However, the majority still found it difficult, implying that linguistic expression is still developing. This association between explanation and problem-solving measures has been found in previous research. For example when children were prompted to explain what they observed (i.e., how a toy worked), they explored inconsistent outcomes and engaged in hypothesis testing strategies (Legare, 2012); and focused more on causal properties than on perceptual features of the evidence (Legare and Lombrozo, 2014; Walker et al., 2014). It has been argued that explaining promotes learning because it requires one to integrate evidence with prior beliefs (Lombrozo, 2006) and hence placing observations in the context of a larger and coherent framework (Wellman and Liu, 2007). Therefore, if some children were engaging in self-explanation while trying to solve this task, this could explain why they performed better than their peers. Further work could test this notion by prompting children to seek an explanation for the sounds, to see if this improves performance in 4- and 5- year-olds, which, while above chance, was not by any means at ceiling level.

To conclude, this work contributed to the developmental and comparative literature by introducing a novel paradigm that contrasts learning in a causal and an arbitrary context without the need for verbal instruction. We argue that our results are in line with previous suggestions that by 4-years of age, children are able to use evidence to detect unseen causes. It is possible that this stems from a tendency to seek causal explanations even in the absence of instruction to do so. Studies on exploratory play in young children conducted by Schulz and colleagues provide similar evidence that 4-year-old children are actively seeking out causal explanations. For instance, when provided with ambiguous information about how a toy worked, they spontaneously explored the toy’s function rather than playing with a new toy as opposed to when the function was unambiguous (Schulz and Bonawitz, 2007); and they also conducted informative interventions (Cook et al., 2011). However, further work is needed to determine the reasons for the negative results found with younger children and chimpanzees. One possible avenue for future research will be the use of visual cues instead of auditory cues with a similar paradigm. This might improve performance of preschoolers and chimpanzees by lowering the cognitive load associated with tracking and remembering transient auditory cues.

## Data Availability Statement

All datasets generated for this study are included in the Supplementary Material.

## Ethics Statement

The studies involving human participants were reviewed and approved by University of St Andrews Teaching and Research Ethics Committee. Written informed consent to participate in this study was provided by the participants’ legal guardian/next of kin. The animal study was reviewed and approved by University of St Andrews School of Psychology and Neuroscience Ethics Committee.

## Author Contributions

ZC performed the data collection, statistical analysis and wrote the first draft of the manuscript. All authors contributed to the design of the studies, revisions, read and approved the submitted version.

## Conflict of Interest

The authors declare that the research was conducted in the absence of any commercial or financial relationships that could be construed as a potential conflict of interest.
