# Supplementary material for: Inferring Unseen Causes: Developmental and Evolutionary Origins
Source: Front Psychol. 2020 May 6;11:872. doi: 10.3389/fpsyg.2020.00872 (PMC7218093; doi:10.3389/fpsyg.2020.00872)
Supplement: Supplementary file 1 [file Data_Sheet_1.docx]

Supplementary Material

# Supplementary Tables

### Table S1. Output of GLMM 01 Experiment 1: Correct choices across the different conditions

|  | Estimate | SE | Χ² | DF | P | 95% CI | |
| --- | --- | --- | --- | --- | --- | --- | --- |
| (Intercept) | -0.194 | 0.150 |  |  | 0.197 | -0.502 | 0.083 |
| Age^1^: four | 0.696 | 0.203 |  |  | 0.001 | 0.315 | 1.117 |
| Age^1^: five | 0. 432 | 0.201 |  |  | 0.032 | 0.038 | 0.826 |
| Age^1^: six | 0.540 | 0.199 |  |  | 0.007 | 0.156 | 0.931 |
| Condition^2^ | 0.182 | 0.200 |  |  | 0.364 | -0.180 | 0.573 |
| Sex^3^ | 0.180 | 0.101 | 3.168 | 1 | 0.075 | -0.016 | 0.391 |
| Trial number^4^ | 0.115 | 0.043 | 6.996 | 1 | 0.008 | 0.032 | 0.196 |
| Age^1^ x Condition^2^ |  |  | 8.709 | 3 | 0.033 |  |  |
| Age^1^: four x Condition^2^:arbitrary | -0.812 | 0.285 |  |  | 0.004 | -1.348 | -0.315 |
| Age^1^: five x Condition^2^:arbitrary | -0.555 | 0.284 |  |  | 0.051 | -1.103 | -0.035 |
| Age^1^: six x Condition^2^:arbitrary | -0.293 | 0.283 |  |  | 0.301 | -0.845 | 0.241 |

Notes: Reference categories: ^1^three, ²causal, ^3^female. Covariates were z-transformed to a mean of zero and a standard deviation of one; mean (sd) of the original variable were ^4^10.50 (5.77).

### Table S2. Output of GLMM 02 Experiment 1: Correct choices based on children’s explanations

|  | Estimate | SE | Χ² | DF | P | 95% CI | |
| --- | --- | --- | --- | --- | --- | --- | --- |
| (Intercept) | 1.583 | 0.244 |  |  | 0.000 | 1.143 | 2.075 |
| Explanation^1^ | -1.356 | 0.235 | 24.899 | 1 | 0.000 | -1.858 | -0.944 |
| Sex^2^ | -0.343 | 0.231 | 2.240 | 1 | 0.135 | -0.803 | 0.104 |
| Trial number^3^ | 0.267 | 0.124 | 4.409 | 1 | 0.036 | 0.027 | 0.530 |

Notes: Reference categories: ^1^correct, ^2^female. Covariates were z-transformed to a mean of zero and a standard deviation of one; mean (sd) of the original variable were ^3^10.50(5.77).

### Table S3. Output of GLMM 01 Experiment 2: Correct choices across the different sound orders

|  | Estimate | SE | Χ² | DF | P | 95% CI | |
| --- | --- | --- | --- | --- | --- | --- | --- |
| (Intercept) | 0.583 | 0.277 |  |  | 0.036 | 0.081 | 1.134 |
| Condition^1^ | 0.013 | 0.308 | 1 | 0.002 | 0.966 | -0.557 | 0.589 |
| Sex^2^ | -0.041 | 0.308 | 1 | 0.018 | 0.895 | -0.665 | 0.519 |
| Trial number^3^ | 0.056 | 0.094 | 1 | 0.359 | 0.549 | -0.122 | 0.237 |

Notes: Reference categories: ^1^causal order, ^2^female. Covariates were z-transformed to a mean of zero and a standard deviation of one; mean (sd) of the original variable were ^3^10.50(5.77).

### Table S4. Output of GLMM 01 Experiment 3: Correct choices in unfamiliar boxes causal condition with chimpanzees.

|  | Estimate | SE | Χ² | DF | P | 95% CI | |
| --- | --- | --- | --- | --- | --- | --- | --- |
| (Intercept) | -0.013 | 0.082 |  |  | 0.870 | -0.148 | 0.176 |
| Age^1^ | -0.014 | 0.082 | 0.029 | 1 | 0.865 | -0.166 | 0.139 |
| Session number^2^ | 0.007 | 0.082 | 0.007 | 1 | 0.932 | -0.163 | 0.172 |
| Trial number^3^ | 0.102 | 0.083 | 1.403 | 1 | 0.236 | -0.051 | 0.273 |

Notes: Covariates were z-transformed to a mean of zero and a standard deviation of one; mean (sd) of the original variable were ^1^27.83 (11.86), ^2^5.5 (2.87), ^3^5.5(2.87).

### Table S5. Output of GLMM 02 Experiment 3: Correct choices in familiar boxes causal and arbitrary conditions with chimpanzees.

|  | Estimate | SE | Χ² | DF | P | 95% CI | |
| --- | --- | --- | --- | --- | --- | --- | --- |
| (Intercept) | -0.007 | 0.104 |  |  | 0.944 | -0.212 | 0.196 |
| Condition^1^:arbitrary_familiar | 0.083 | 0.125 | 1 | 0.446 | 0.504 | -0.171 | 0.324 |
| Experience^2^:none | 0.141 | 0.140 | 1 | 1.013 | 0.314 | -0.130 | 0.419 |
| Age^3^ | -0.017 | 0.071 | 1 | 0.054 | 0.816 | -0.163 | 0.128 |
| Session number^4^ | 0.025 | 0.060 | 1 | 0.177 | 0.674 | -0.099 | 0.149 |
| Trial number^5^ | -0.048 | 0.060 | 1 | 0.639 | 0.424 | -0.170 | 0.068 |

Notes: Reference categories: ^1^causal_familiar, ²none. Covariates were z-transformed to a mean of zero and a standard deviation of one; mean (sd) of the original variable were ^3^22.64 (11.30), ^4^5.5 (2.87), ^5^5.5(2.87).

### Table S6. Output of GLMM 01 Experiment 4: Correct choices in shaken boxes with children.

|  | Estimate | SE | Χ² | DF | P | 95% CI | |
| --- | --- | --- | --- | --- | --- | --- | --- |
| (Intercept) | 0.277 | 0.347 |  |  | 0.424 | -0.364 | 0.964 |
| Age^1^: four | 0.819 | 0.433 |  |  | 0.058 | -0.026 | 1.658 |
| Age^1^: five | 1.852 | 0.479 |  |  | 0.000 | 0.958 | 2.810 |
| Age |  |  | 15.257 | 2 | 0.000 |  |  |
| Sex^2^ | -0.138 | 0.366 | 0.142 | 1 | 0.706 | -0.813 | 0.634 |
| Trial number^3^ | -0.158 | 0.142 | 1.211 | 1 | 0.271 | -0.458 | 0.112 |

Notes: Reference categories: ^1^three, ²female. Covariates were z-transformed to a mean of zero and a standard deviation of one; mean (sd) of the original variable were ^1^4.46 (0.83), ^3^5.5 (2.88).

### Table S7. Output of GLMM 02 Experiment 4: Correct choices in shaken boxes with chimpanzees.

|  | Estimate | SE | Χ² | DF | P | 95% CI | |
| --- | --- | --- | --- | --- | --- | --- | --- |
| (Intercept) | -0.189 | 0.148 |  |  | 0.202 | -0.489 | 0.091 |
| Age^1^ | -0.019 | 0.062 | 0.092 | 1 | 0.761 | -0.145 | 0.108 |
| Session number^2^ | 0.030 | 0.022 | 1.992 | 1 | 0.159 | -0.012 | 0.072 |
| Trial number^3^ | 0.049 | 0.086 | 0.327 | 1 | 0.565 | -0.113 | 0.226 |

Notes: Covariates were z-transformed to a mean of zero and a standard deviation of one; mean (sd) of the original variable were ^1^22.81 (11.32), ^2^5.43 (2.85), ^3^5.50(2.87).

### Table S8. Output of GLMM 01 Experiment 5: Correct choices in shaken boxes with 3-year olds.

|  | Estimate | SE | Χ² | DF | P | 95% CI | |
| --- | --- | --- | --- | --- | --- | --- | --- |
| (Intercept) | 0.581 | 0.325 |  |  | 0.074 | -0.039 | 1.182 |
| Question type^1^:causal | 0.504 | 0.341 | 2.112 | 1 | 0.146 | -0.117 | 1.115 |
| Sex^2^ | -0.575 | 0.329 | 3.042 | 1 | 0.081 | -1.236 | 0.040 |
| Trial number^3^ | 0.062 | 0.117 | 0.284 | 1 | 0.594 | -0.177 | 0.289 |

Notes: Reference categories: ^1^noncausal, ²female. Covariates were z-transformed to a mean of zero and a standard deviation of one; mean (sd) of the original variable were ^3^5.50(2.88).
